# Obesity-Related Differences in Amygdala and Hippocampus Volume and Metabolism in Major Depressive Disorder: Implications for Antidepressant Treatment

**DOI:** 10.21203/rs.3.rs-6257703/v1

**Published:** 2025-05-08

**Authors:** Karen Lin, Karin Hasegawa, Vindhya Rapelli, Jie Yang, Ramin Parsey, Christine DeLorenzo

**Affiliations:** Cornell University; Stony Brook University

## Abstract

The link between obesity and depression is bidirectional and antidepressant efficacy is reduced in obese individuals. To probe this mechanism, anthropometric measures (body mass index [BMI] and waist circumference [WC]) from 85 participants (n = 56 females, n = 29 males) with Major Depressive Disorder (MDD) were acquired. Participants then received simultaneous positron emission tomography (PET)/magnetic resonance imaging (MRI) before and after treatment with a double-blind randomized trial of escitalopram. Linear mixed models were used to examine the association of continuous and categorical anthropometric measures with pretreatment amygdala and hippocampus volumes and metabolism, controlling for covariates. Similar models assessed the association with percent change in brain volume and metabolism with treatment, controlling for treatment type. Pretreatment, obesity was significantly positively associated with amygdala (p = 0.0160 and 0.0154, for WC and BMI, respectively) and hippocampus (p = 0.0221 for BMI) volume, but not metabolism. With treatment, each additional inch of WC (or each lb/in^2^ of BMI) was associated with a −0.38% (or −0.26%) treatment-induced change in amygdala volume. In fact, past 37.29 in (border of increased risk for males, substantially increased risk for females) or 30.63 lb/in^2^ (obese range), the percent change in amygdala volume with treatment shifts from positive (increasing) to negative (decreasing). Further, for every 1 lb/in^2^ in BMI, there was a 1.03% (amygdala) or 0.99% (hippocampus) increase in metabolism with treatment. This multimodal study is the first to suggests that obesity-related factors may alter the structure and metabolism of the amygdala and hippocampus, potentially reducing antidepressant effectiveness in MDD.

## Introduction

According to the World Health Organization (WHO), depression is the most common mental illness, affecting 280 million individuals worldwide.^1^ Contributing to depression’s impact, there is broad variation in antidepressant therapy response,^2^ leading to unresolved depression, impairing an individual’s quality of life and heightening mortality.^3^ One factor that may cause poor treatment outcomes is obesity.^2^ For example, obese individuals demonstrate unfavorable treatment outcomes including resistance to selective serotonin reuptake inhibitors (SSRIs).^4–6^

Depression and obesity are bidirectionally related^7^ -- a meta-analysis found that depressed people had 58% increased risk of becoming obese and people with obesity have a 55% increased risk of developing depression.^8^ Despite the associations between obesity, depression and poor treatment outcomes, the biological underpinnings of these relationships are not understood, though they may lie in the brain. Located in the temporal lobe, the amygdala and the hippocampus of the limbic system play a role in emotional processing and episodic memory, respectively, and together aid in the formation of long-term memories of significantly emotional episodes.^9^ They are known to be separately and collectively affected in major depressive disorder (MDD) and by SSRIs as well as obesity.^10^

Hippocampal volume is often lower in depressed individuals compared to nondepressed controls.^11–20^ Most meta-analyses also found amygdala volumes of patients with depression to be smaller than controls^21–23^ although one found no differences.^24^ Some studies also suggest the amygdala may be enlarged during the first episode of depression^25,26^ with decreased volume in subsequent years.^27^ In addition to structure, studies have also examined hippocampal and amygdala function. Depression severity has been shown to be negatively correlated with hippocampal metabolism,^28^ while patients with MDD have exhibited significantly increased activity and metabolism in the amygdala compared to nondepressed controls.^29–33^

Similarly, obese individuals exhibit smaller volumes of the hippocampus^34–37^ while conflicting results have been observed for volume differences in the amygdala, with some studies showing grey matter volume reduction in obese individuals when compared to non-obese individuals^38,39^ and others showing a positive association between BMI^40^ or poor dietary impulse control^41^ and amygdala volume. However, a high fat diet and consumption of ultra-processed foods is associated with reduced volumes of amygdala and hippocampus.^42,43^ Also similar to depression, amygdala activity was positively associated with visceral adiposity at baseline and subsequent gain in adiposity;^44^ however hippocampal metabolism may be higher in obesity as well, since diminished glucose metabolism in the hippocampus was found after bariatric surgery.^45^

Altered brain volumes and activity in both depressed and obese individuals may be attributed to numerous factors, including altered hypothalamic-pituitary-adrenal (HPA) axis function, through multiple pathways. One such pathway could involve inflammation. Excess adiposity increases circulating free fatty acids reaching the brain, causing local inflammation and neurodegeneration of the hypothalamus, in the HPA axis, resulting in disruption to the hippocampus and amygdala.^46,47^ Depression is also associated with increased inflammation.^48–50^

Another common HPA related pathway is through increased glucocorticoids, and reduced brain-derived neurotrophic factors (BDNF). People with depression report a higher rate of stressful life events.^51^ Life stress induces a response in the HPA axis, leading to the release of corticotropin-releasing factor (CRF), which leads to the release of adrenocorticoid tropic hormones. This pathway then signals the adrenal cortex to produce and release glucocorticoids.^52^ In obese individuals, increased adipose tissue can also trigger this glucocorticoid production pathway.^53^ Recent studies suggest that glucocorticoids decrease the transcription and secretion of BDNF,^54^ which may be responsible for reduced hippocampal and amygdala volumes. Overweight and obese individuals have lower levels of BDNF, which deregulates food intake.^55,56^ Calorically high, obesity-inducing diets can further reduce BDNF levels in the brain and can impair neural plasticity and neurogenesis.^55^ The neurotrophic hypothesis of depression posits that a reduction in the concentration of BDNF plays a critical role in depression as well.^57^

SSRIs normalize HPA activity, resulting in lower glucocorticoid levels, in rats.^58^ Although their exact mechanism is unknown, one explanation for the time required for clinical efficacy is due to SSRI-induced production of BDNF, which leads to the formation of new neurons in the hippocampus^59,60^ and amygdala.^24^ Consequently, some studies have found long term antidepressant treatment increases the volume of the hippocampus and amygdala while unmedicated patients with MDD experienced a reduction in the volume of these regions over the same period.^24,61^ Volume change of the hippocampus and amygdala may be needed for antidepressant response.^62,63^ (However, some studies have found no significant changes in hippocampus volume following antidepressant treatment.^64,65^) Additionally, SSRIs were found to decrease activation of the amygdala and hippocampus in patients with MDD.^31,66–68^

Combining the accumulating evidence above suggests that SSRI mechanisms may be affected by obesity, potentially accounting for reduced efficacy. Therefore, in this study, we examine effects of depression *and* obesity on brain structure/function using anthropometric measures and multimodal (positron emission tomography and magnetic resonance imaging) neuroimaging before and after treatment. We hypothesize that, in depressed individuals, pretreatment waist circumference is negatively associated with hippocampus and amygdala volume and metabolism. As the effects of obesity may attenuate SSRI induced changes, we further hypothesize that greater pretreatment waist circumference will be associated with a smaller percent increase in hippocampus and amygdala volume and a smaller reduction in amygdala and hippocampus metabolism following treatment, which could provide insight into the reduced antidepressant treatment efficacy in obesity.

Obesity is typically assessed through body mass index (BMI). However abdominal obesity, also known as central or visceral obesity, assessed by waist circumference, has shown a stronger association with depression than BMI.^69,70^ This is possibly due to differences in the type of adipose tissue near the abdomen. White adipose tissue (WAT) can be divided into two subdivisions, visceral or subcutaneous, with distinct structural and metabolic characteristics.^71^ Visceral WAT exhibits greater metabolic activity, produces higher systemic inflammation and has direct access to the portal circulation, unlike subcutaneous WAT.^69,72^ In addition to depression, the accumulation of visceral WAT is associated with metabolic disorders, including dyslipidemia, hypertension and insulin resistance, and cardiovascular disease.^71,73^ Therefore, waist circumference is used as the primary assessment of obesity in this study.

## Methods

The study “Advancing Personalized Antidepressant Treatment Using PET/MRI” is registered on ClinicalTrials.gov (NCT02623205) and approved by the Institutional Review Board of Stony Brook University (see CONSORT diagram in^74^). Inclusion criteria consisted of: 18 years old or older and the ability to sign informed consent. Potential participants were excluded if they had a lifetime history of bipolar disorder, a significant active physical illness, significant neurological deficits, current psychosis, or a high potential for excessive substance use during the study period. Additional exclusion criteria included participants receiving successful antidepressant treatment, who had electroconvulsive therapy within six months, medical contraindications to escitalopram (study drug), such as failed escitalopram therapy of appropriate dose and duration in the past, or contraindications to MRI or PET imaging, such as pregnancy or breastfeeding.

All participants were assessed via Structured Clinical Interview for DSM-IV (SCID-IV) and were verified by a trained rater as having been diagnosed with MDD with a score of 22 or higher on the Montgomery–Åsberg Depression Rating Scale^75^ (MADRS). All participants were antidepressant medication-free for three weeks prior to the study, either having completed medication washout (for ineffective medication) or by enrolling as psychotropic medication free. PET/MRI data from this cohort has been published in previous studies (e.g.^74,76,77^). None have examined the relationship between obesity and PET/MRI measures as conducted in this work.

Height, weight and waist circumference were measured at the participant screening visit. Using their waist circumferences, participants were grouped into three categories based on WHO cutoffs^78^: low risk (≤ 94 cm for males and ≤ 80 cm for females), increased risk (> 94 to 102 cm for males and > 80 to 88 cm for females) and substantially increased risk (> 102 cm for males and > 88 cm for females). Based on BMI, participants were classified as underweight (< 18.5 lb/in^2^), healthy weight (18.5 to 25 lb/in^2^), overweight (25 to 30 lb/in^2^) and obese (> 30 lb/in^2^).^79^

Simultaneous PET/MRI imaging was acquired on a 3T Siemens Biograph mMR (Siemens, Erlangen, Germany) with a 12-channel head coil for 60 min pre and post treatment. The Hamilton Depression Rating Scale (HDRS-17) was assessed within approximately one week of imaging. Treatment was initiated following imaging. Through a double-blind design, participants were randomized to treatment with either placebo or escitalopram. In this cohort, neurobiological changes quantified by FDG-PET were not different across participants who achieved depression remission using either placebo or SSRI.^80^ As this is consistent with multiple previous studies,^81–85^ escitalopram and placebo subgroups were examined together, with treatment included as a covariate.

### Magnetic Resonance Imaging (MRI)

A magnetization-prepared rapid gradient-echo (MP-RAGE) T1-weighted structural image was acquired, simultaneously with the PET imaging, with the following parameters: TR = 2300ms, TE = 3.24ms, flip angle = 9 degrees, IPAT GRAPPA factor 2, FOV = 223×210×195mm, bandwidth = 220 Hz/Px, echo spacing = 7.8ms, voxel size = 0.87×0.87×0.87mm. T1 structural images were processed through the egmentation pipeline of Freesurfer 5.3.0 (http://surfer.nmr.mgh.harvard.edu) to automatically extract the amygdala and hippocampus from the Desikan-Killiany atlas.^86,87^

### Positron Emission tomography (PET)

Raw listmode PET data, collected for 60 min, were reconstructed offline using Siemens’ e7 Tools software and a CT-like Boson MR-based attenuation map.^88,89^ Frames were corrected for motion^90^ and co-registered to the MRI. The regional delineations were transferred to the PET images through the co-registration. Metabolic rate of glucose uptake (MRGlu) was estimated from the Patlak graphical using the time activity curve while correcting for blood glucose and the lumped constant, using Simultaneous Estimation and a single venous sample, as previously described.^74^

Of the 85 participants who received pretreatment imaging, 12 participants’ PET measures were not used because they exhibited a > 20% change in glucose during the pretreatment imaging (n = 11) or due to diagnosis of diabetes (n = 1, removed from all PET analyses). The Freesurfer delineation of the hippocampus from one participant’s pretreatment MRI was misaligned and therefore was not used in the volumetric analysis. Additionally, 12 participants’ post-treatment PET measures were not used because the participants did not complete the treatment protocol (n = 7), or complete posttreatment imaging (n = 2, 1 completed MRI only) or they exhibited a > 20% change in glucose during the post-treatment PET imaging (n = 3). (Note: as one participant exhibited a > 20% change in glucose during the imaging both pre and post treatment, this left 62 participants with useable pre-and post-PET imaging.) Of the 77 participants receiving post-treatment MRI imaging, one participant’s MRI measures could not be used due to excessive motion. In addition, 9 participants had missing waist circumference measurements, and one participant had missing weight information preventing BMI calculations. For three participants, self-reported weight was used instead of that measured at screening.

## Statistical Analysis

Linear mixed models were utilized to examine the association between (or differences across levels of, in the case of categorical measurement) pretreatment waist measurement and (in):

- pretreatment amygdala / hippocampus volume after controlling for sex, age, age^2^ (to account for nonlinear effects) and pretreatment total brain volume,
- pretreatment amygdala / hippocampus metabolism after controlling for sex, age, and age^2^.

As a secondary analysis, these models were repeated with BMI.

Similar linear mixed models were utilized to examine the association between (or differences across levels of, in the case of categorical measurement) pretreatment waist circumference (in)

- percent change in amygdala / hippocampus volume
- percent change in amygdala / hippocampus metabolism,

both after controlling for treatment type. Percent change was calculated using post-treatment value minus pre-treatment value divided by pre-treatment value. The same methods were utilized for BMI.

For each linear mixed model, a covariance structure was utilized to model the correlated measures from the amygdala and hippocampus. Final dependence structure was chosen from Compound Symmetry and Unstructured based on the Akaike Information Criteria (AIC).

Multiple linear regression models were utilized to examine the relationship between pretreatment depression severity and pretreatment waist measurement or BMI (either as continuous or categorical), both after controlling for sex and age.

All the statistical analysis was performed using SAS 9.4 and significance level was set at 0.05 (SAS Institute Inc., Cary, NC).

## Results

Participant information is provided in Table 1. According to waist circumference, 27 (35.53%) individuals have substantially increased risk for a metabolic disorder, 14 (18.42%) have increased risk, and 35 (46.05%) individuals are categorized as low risk. According to their BMI, 23 (27.38%) individuals are categorized as obese, 22 (26.19%) are overweight, 34 (40.48%) are healthy weight, and 5 (5.95%) are underweight.

### Relationship between Continuous Values of Waist Circumference / BMI and Pretreatment Neurobiology

As displayed in Table 2, pretreatment waist measurement had a significantly positive association with pretreatment amygdala volume (estimated coefficient=16.62, 95%, confidence interval [CI]: 3.18–30.05, p-value=0.016) but not with pretreatment hippocampus volume (p-value=0.201). Pretreatment waist measurement was not significantly associated with pretreatment amygdala or hippocampus metabolism (both p-values>0.05). Pretreatment BMI had statistically significant positive associations with both pretreatment amygdala and hippocampus volumes (amygdala: estimated coefficient=15.21, 95% CI: 2.99–27.44, p-value=0.015; hippocampus: estimated coefficient=29.72, 95% CI: 4.39–55.06, p-value=0.022). There was no statistically significant association between pretreatment BMI and pretreatment amygdala or hippocampus metabolism (both p-values>0.05).

For completeness, the relationships between pretreatment waist measurement / BMI and pretreatment depression severity were examined and no significant associations were found (all p-values>0.05, data not shown).

### Relationship between Waist Circumference / BMI and Percent Change in Neurobiology with Treatment

**Figure 1:**
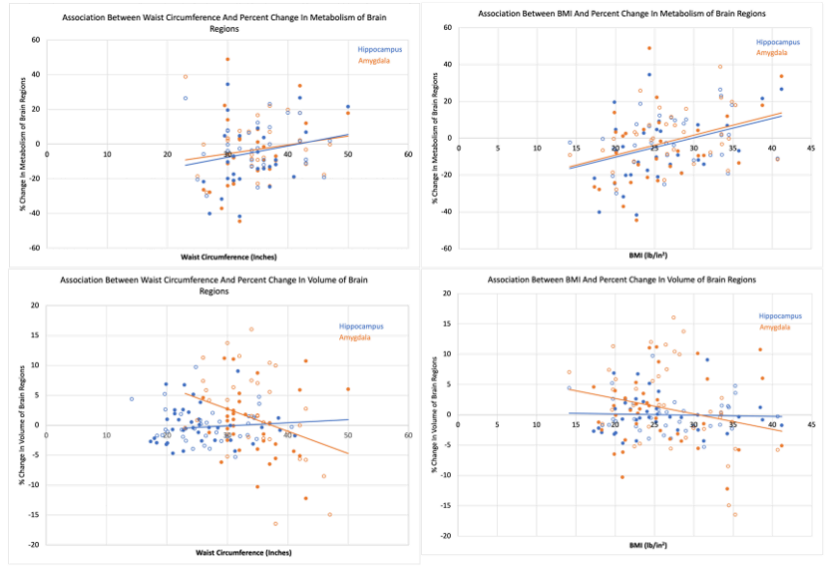
Associations Between Waist Circumference and BMI and Percent Change in Brain Region Volume and Metabolism with treatment. Solid circles indicate escitalopram and open circles indicate placebo treatment. Regression lines are shown for each brain region.

Both pretreatment waist measurement and pretreatment BMI had statistically significant negative associations with the percent change in amygdala volume with treatment (waist measurement: estimated coefficient=−0.38, 95% CI: −0.65−-0.11, p-value=0.006; BMI: estimated coefficient=−0.26, 95% CI: −0.51- −0.01p-value=0.043) but not with percent change in hippocampus volume with treatment (both p-values>0.05, Figure 1 and Table 2). Pretreatment waist measurement was not significantly associated with percent change in amygdala and hippocampus metabolism with treatment (both p-values>0.05, Table 2). Pretreatment BMI had statistically significant positive associations with both the percent change in amygdala and hippocampus metabolism with treatment (amygdala: estimated coefficient=1.03, 95% CI: 0.33–1.73, p-value=0.005; hippocampus: estimated coefficient=0.99, 95% CI: 0.30–1.69, p-value=0.006, Table 2).

There was no statistically significant association between pretreatment waist circumference or BMI and percent change in depression severity (both p-values>0.05, results not shown).

### Relationship between Categories of Waist Circumference / BMI and Pretreatment Neurobiology

As shown in Table 3, pretreatment amygdala and hippocampus volumes were significantly different across some pre-treatment waist measurement categories (amygdala: low vs substantially increased risk: estimated difference=−246.34, 95% CI:−400.74−-91.94, p-value=0.002; increased vs substantially increased risk: estimated difference=−364.04, 95% CI: −566.58−-161.50, p-value=0.001; hippocampus: increased risk vs substantially increased risk: estimated difference=−568.75, 95% CI:−1035.20−-102.31, p-value=0.018). Similar to the continuous analysis, there were no significant differences in pretreatment amygdala and hippocampus metabolism across waist circumference categories (all p-values>0.05).

Also similar to the continuous analysis, pretreatment amygdala and hippocampus volumes had significant differences across pre-treatment BMI categories (amygdala: healthy vs obese: estimated difference=−221.13, 95% CI:−394.51−-47.75, p-value=0.013; overweight vs obese: estimated difference=−223.83, 95% CI: −420.89−-26.77, p-value=0.027; hippocampus: healthy vs obese: estimated difference=−428.98, 95% CI: −795.12−-62.84, p-value=0.022,). There were no significant differences in pretreatment amygdala and hippocampus metabolism across BMI categories (all p-values>0.05).

Neither pre-treatment waist measurement category nor BMI category was significantly associated with pretreatment depression severity (all p-values>0.05, results not shown).

### Relationship between Categories of Waist Circumference / BMI and Percent Change in Neurobiology with Treatment

Mimicking the continuous analysis, percent change in amygdala volume (Table 3) was significantly different across pretreatment waist measurement categories when compared to the substantially increased risk group (low vs substantially increased risk: estimated difference=4.94, 95% CI: 1.79–8.09, p-value=0.003; increased vs substantially increased risk: estimated difference=8.45, 95% CI: 4.34–12.57, p-value=0.0001). Similarly, percent change in amygdala volume was significantly different across pretreatment BMI categories when compared to the obese group (healthy vs obese: estimated difference=4.08, 95% CI: 0.42–7.74, p-value=0.029; overweight vs obese: estimated difference=6.18, 95% CI: 2.18–10.19, p-Page 10/19 value=0.003), except for underweight vs obese group (p-value>0.05). Also, while percent change in amygdala and hippocampus metabolism was not significantly different across categories of pretreatment waist measurement (all p-values>0.05), there were significant differences in both percent change in amygdala and hippocampus metabolism between underweight and obese individuals (amygdala: estimated difference=−22.64, 95% CI:−41.29−-3.99, p-value=0.018; hippocampus: estimated difference=−19.75, 95% CI:- 38.40–1.10, p-value=0.038).

There was no statistically significant difference in change of depression severity with treatment across categories of pretreatment waist circumference or BMI (all p-values>0.05, results not shown).

## Discussion

In this study, two anthropometric measures were used to evaluate body composition or adiposity in relation to neurobiology in individuals with MDD before and after treatment. Previous studies have shown neurobiological correlates of obesity, depression and antidepressant treatment. This study is the first, to our knowledge, to examine the interplay of all three, and potentially uncover a mechanism for reduced efficacy of antidepressant treatment in obese individuals.

Waist circumference, measuring visceral fat, is one among five diagnostic criteria for diagnosing metabolic syndrome.^91,92^ However, BMI is associated with body fat and a measure that is well-established in the literature for measuring obesity due to its practicality and ease of collection. While the BMI and waist circumference showed the same or similar relationships to brain biology in this work, the exception was percent change in metabolism with treatment, which only differed across BMI measures. Analysis treating BMI as a categorical variable provides insight into this, suggesting it was driven by difference between the underweight participants and the obese participants. As there were only five underweight participants, this should be confirmed in a larger sample. However, this is consistent with the fact that waist circumference does not categorize underweight individuals and is generally not a measure of overall obesity, but rather metabolic disease and risk.^93–95^ Because of this, the BMI measure would be more sensitive to differences that occur in underweight individuals.

### Relationship between Waist Circumference / BMI and Pretreatment Neurobiology

Both waist circumference and BMI were correlated with greater amygdala volume. We hypothesized an inverse correlation due to diffuse volume reductions observed in obesity and in high fat diets.^42,43^ However, diets were not assessed in this work and a positive association between BMI and amygdala volume has been previously shown.^40^ The categorical analysis provides additional insight into these differences. Specifically, significant differences were only found between low / increased risk and substantially increased risk (but not between low and increased risk). Similarly, differences were found between healthy / overweight and obese (but not between under / healthy weight and obese). This suggests that these differences may only be observable with inclusion of participants with higher levels of waist circumference / BMI.

Studies have previously found smaller hippocampal volumes separately in depression and obesity. Again, contrary to our hypothesis, in this cohort of participants with MDD, pretreatment BMI was found to have a significant positive association with pretreatment hippocampal volume. Analysis treating BMI as a categorical variable suggests the hippocampal association is driven only by difference between healthy weight and obese groups. Analysis treating waist circumference as a categorical variable also found differences across in pretreatment hippocampal volume only between increased risk and substantially increased risk groups. When combined with the lack of association between continuous waist circumference and hippocampal volume, this suggests hippocampal differences are observable over specific ranges of waist circumference.

Notably, the associations between waist circumference/BMI and brain volume exist even though no association between waist circumference/BMI and depression severity was found, suggesting that depression severity did not confound these relationships.

In a previous analysis of this cohort, we hypothesized that reduced hippocampal volume occurred in MDD, in part, due to a hyperactive hypothalamus and exposure to glucocorticoids but found no significant association between hypothalamus activity (or percent change in hypothalamus activity with treatment) and hippocampus volume (or percent change in hippocampal volume with treatment).^96^ Taken together with this work, these findings suggest that, in this cohort, HPA axis hyperactivity (e.g. due to obesity) is not associated *reduced* amygdala and hippocampal volume. However, it is possible that there is an inflammation-induced *hypertrophy* of these regions in response to obesity related factors.

Neither pre-treatment waist circumference nor BMI was associated with metabolism (hippocampus or amygdala). This lack of correlation may indicate that underlying variability plays a role in regional metabolism including differences in genetic metabolic regulation, neuroendocrine sensitivity, and lifestyle, diet or environmental factors. Therefore, associations across participants may be more challenging to identify than within-participant associations (described in the next section).

### Relationship between Waist Circumference / BMI and Percent Change in Neurobiology with Treatment

Though this study involved both SSRIs and placebo, the percent change in volume and metabolism were not significantly different across treatments (data not shown). Importantly, consistent othe studies,^97,98^ there was significant variability in treatment response across the cohort (Table 1). The purpose of this study is to understand how obesity affects this variability.

Both pretreatment waist circumference and BMI showed a significant negative association with percent change in amygdala volume. For every 1 inch increase in waist circumference or 1 lb/in^2^ increase in BMI, there was a 0.38% or a 0.26%, respectively, decrease in percent change of amygdala volume with treatment. In fact, past 37.29 in (border of increased risk for males, substantially increased risk for females) and 30.63 lb/in^2^ (obese range), the regression fit line suggests the percent change in amygdala volume with treatment crosses the x-axis and becomes negative (Fig. 1 bottom row). Consistently (with the regression analysis and the pretreatment analysis), the categorical analysis shows these differences were found between low / increased risk and substantially increased risk (but not between low and increased risk) and between healthy / overweight and obese (but not between under / healthy weight and obese). This could indicate that, at certain higher levels of obesity, antidepressant action is affected due to the interaction of obesity with treatment mechanism at the amygdala. And specifically, at higher obesity levels attenuated increases (or even decreases) in amygdala volume with treatment occur.

When combined with the pretreatment findings in this cohort (that obesity is associated with higher pretreatment amygdala volume), this suggests that the mechanisms driving the initial enlargement of the amygdala in obesity (e.g., inflammation) may differ from those involved in treatment-related volume changes (e.g., neurogenesis) and could even hinder these changes.

No significant relationship between waist measurement / BMI and percent change in hippocampal volume with treatment was found. This is consistent with the fact that the hippocampus is more susceptible to neurogenesis-dependent changes than the amygdala^99,100^ and highlights both that obesity/treatment interactions may be region specific and the importance of the amygdala in this interaction.

As antidepressant treatment has been shown to decrease amygdala and hippocampal activity, metabolic results also indicate a counteractive effect of obesity, as hypothesized. In fact, the percent change in metabolism from negative to positive occurs at 41.50 inches (substantially increased risk) / 29.77 lb/in^2^ (obese range is > 30) for the hippocampus and 40.90 inches / 28.46 lb/in^2^ for the amygdala. Further, for every 1 lb/in^2^ increase, there was a 1.03% or a 0.99% increase in percent change of amygdala or hippocampus metabolism, respectively. Across the range of BMIs in this study (14.2–41.2 lb/in^2^), this would result in a significant (27–28%) difference in treatment induced effects on the amygdala / hippocampus metabolism.

### Strengths & Limitations

While BMI and waist circumference are non-invasive, cheap and easy to measure, the cutoffs and categorization of individuals does not sufficiently capture the nuance of individual body composition, especially for individuals at the margin between two categories. The gold standard for assessing body composition, the dual-energy x-ray absorptiometry (DXA), was not utilized in this study and could be a potential limitation. In addition, weight was not tracked throughout treatment, but observations indicated no significant changes. It is also important to note that the relatively young median age of participants in this study, 23.6, limits the generalizability of the findings to the broader population. However, this younger cohort remains an important group to investigate as early intervention through reducing waist circumference or BMI could yield benefits for improving antidepressant treatment efficacy in this demographic.

The strength of this study includes multiple anthropometric measures such as waist circumference and BMI and simultaneous PET and MRI imaging in a large sample size. Also, approximately half of the participants were overweight and obese individuals (45 out of 84, 53.57%) and at increase or substantially increase risk for metabolic disorder (41 out of 76, 53.95%). And, rigorous statistical analyses were used, including the use of covariates treatment type, total brain volume, sex, age, and age^2^.

## Conclusion

This multimodal study is the first to show an association between obesity measures and volume/metabolism in key regions involved in depression- the amygdala and hippocampus. Results indicate that amygdala and hippocampal volume is higher with great levels of obesity, though the hippocampal finding may be sensitive to the ranges evaluated. Further, effects of both active treatment and placebo differed with level of obesity, such that expected effects on volume and metabolism were attenuated. This sheds light on the neurobiological underpinnings of reduced antidepressant response in obesity.

## Figures and Tables

**Table 1:** Participant Demographics and Brain Measures.

Variable	N	Levels	Total
Sex	85	Female	56 (65.88%)
		Male	29 (34.12%)
Age	85		23.60 ± 13.10
BMI (lb/in^2^)	84		25.45 ± 8.13
BMI Category	84	Underweight	5 (5.95%)
		Healthy weight	34 (40.48%)
		Overweight	22 (26.19%)
		Obese	23 (27.38%)
Waist Measurement (in)	76		34.00 ± 7.00
Waist Measurement Category	76	Low risk	35 (46.05%)
		Increased risk	14 (18.42%)
		Substantially increased risk	27 (35.53%)
**Hamilton Depression Rating Scores (HAM-D)**			
Pre-Treatment HAM-D Score	85	18.00 ± 5.00	
Post-Treatment HAM-D Score	78	10.00 ± 9.00	
% Change of HAM-D Score	78	−44.44 ± 44.59	
**Pretreatment Brain Measures**			
Pre-treatment total AMY volume (cm^2^)	85	3183.20 ± 542.50	
Pre-treatment total HIP volume (cm^2^)	84	8494.30 ± 1100.35	
Pre-treatment total AMY metabolism (mg/min*100 ml)	73	3.14 ± 0.66	
Pre-treatment total HIP metabolism (mg/min*100 ml)	73	3.49 ± 0.69	
**Posttreatment Brain Measures**			
Post-treatment total AMY volume (cm^2^)	76	3227.00 ± 482.00	
Post-treatment total HIP volume (cm^2^)	76	8517.00 ± 1044.00	
Post-treatment total AMY metabolism (mg/min*100 ml)	72	3.06 ± 0.55	
Post-treatment total HIP metabolism (mg/min*100 ml)	72	3.34 ± 0.63	
**% Change in Brain Measures**			
% Change of AMY Volume	76	0.92 ± 9.19	
% Change of HIP Volume	75	−0.49 ± 3.61	
% Change of AMY Metabolism	62	−1.26 ± 22.46	
% Change of HIP Metabolism	62	−3.76 ± 22.09	

Note: For categorical variables, percentages across the levels were reported. For continuous variables, median and interquartile range were reported.

AMY: amygdala; BMI: body mass index; HIP: hippocampus

**Table 2:** Associations Between Waist Circumference and BMI and Pretreatment / Percent Change with treatment in Brain Region Volume and Metabolism.

	Waist Measurement (inches)				BMI (lb/in^2^)		
Outcome	Region	Estimated coefficient and 95% CI	P-value*	P-value**	Estimated coefficient and 95% CI	P-value*	P-value**
**Pre-treatment regional brain volume**	AMY	16.62 (3.18, 30.05)	0.0160	0.0538	15.21 (2.99, 27.44)	0.0154	0.0232
	HIP	18.69 (−10.18, 47.55)	0.2009		29.72 (4.39, 55.06)	0.0221	
**Pre-treatment regional brain metabolism**	AMY	−0.005 (−0.026, 0.017)	0.6644	0.0664	−0.011 (−0.030, 0.009)	0.2954	0.0304
	HIP	−0.013 (−0.034, 0.009)	0.2444		−0.018 (−0.038, 0.002)	0.0713	
**% change in regional brain volume**	AMY	−0.38 (−0.65, −0.11)	0.0064	0.0078	−0.26 (−0.51, −0.01)	0.0433	0.1262
	HIP	0.05 (−0.09, 0.19)	0.4584		−0.02 (−0.14, 0.10)	0.7225	
**% change in regional brain metabolism**	AMY	0.47 (−0.36, 1.30)	0.2577	0.2400	1.03 (0.33, 1.73)	0.0048	0.0174
	HIP	0.62 (−0.21, 1.45)	0.1374		0.99 (0.30, 1.69)	0.0061	

* P-values were from Type III T-test for the marginal signifi cance of each effect based on linear mixed models.

** P-values were from Type III F-test for testing the signifi cance of an interaction term based on linear mixed models.

AMY: amygdala; BMI: body mass index; HIP: hippocampus

**Table 3:** Significant Associations Between Categories of Waist Circumference and BMI and Pretreatment / Percent Change with treatment in Brain Region Volume and Metabolism.

	Waist Measurement (inches)					BMI (lb/in^2^)			
Outcome	Level		Estimated difference and 95% CI	P-value*	P-value**	Level	Estimated difference and 95% CI	P-value*	P-value**
**Pre-treatment regional brain volume**	AMY	Low risk vs Substantially increased risk	−246.34 (−400.74, −91.94)	0.0022	0.0036	Healthy weight vs Obese	−221.13 (−394.51, −47.75)	0.0131	0.0793
		Increased risk vs Substantially increased risk	−364.04 (−566.58, −161.50)	0.0006		Overweight vs Obese	−223.83 (−420.89, −26.77)	0.0265	
	Hip	Increased risk vs Substantially increased risk	−568.75 (−1035.20, −102.31)	0.0176		Healthy weight vs Obese	−428.98 (−795.12, −62.84)	0.0222	
**% change in regional brain volume**	AMY	Low risk vs Substantially increased risk	4.94 (1.79, 8.09)	0.0026	0.0016	Healthy weight vs Obese	4.08 (0.42, 7.74)	0.0294	0.0406
		Increased risk vs Substantially increased risk	8.45 (4.34, 12.57)	0.0001		Overweight vs Obese	6.18 (2.18, 10.19)	0.0029	
**% change in regional brain metabolism**	-	-			-	Underweight vs Obese	−22.64 (−41.29, −3.99)	0.0183	0.2699
	-					Underweight vs Obese	−19.75 (−38.40, −1.10)	0.0384		

* P-values were from Type III T-test for testing the signifi cance of the pairwise difference based on linear mixed models.

** P-values were from Type III F-test for testing the signifi cance of an interaction term based on linear mixed models.

AMY: amygdala; BMI: body mass index; HIP: hippocampus
